# A Decade in Periprosthetic Joint Infection Care: Growing Referrals and High Resource Demands at a High-Volume Center

**DOI:** 10.1016/j.artd.2026.102196

**Published:** 2026-10-28

**Authors:** Joshua P. Rainey, Brenna E. Blackburn, Reed C. Noyd, Jessica Hammond, Laura K. Certain, Michael J. Archibeck, Lucas A. Anderson, Christopher L. Peters, Jeremy M. Gililland, Christopher E. Pelt

**Affiliations:** a Department of Orthopaedic Surgery, University of Utah, Salt Lake City, UT; b Department of Internal Medicine, University of Utah, Salt Lake City, UT

**Keywords:** Periprosthetic joint infection, Revision total joint arthroplasty, Tertiary referral center, Healthcare source utilization, Reoperation

## Abstract

**Background:**

The management of periprosthetic joint infection (PJI) requires a multidisciplinary approach that is not feasible in all healthcare settings. This study characterized the evolving burden of PJI referrals at an academic referral center over the past decade.

**Methods:**

A retrospective review was conducted of all referred patients who underwent treatment for hip or knee PJI at a single referral center between 2014 and 2024. Demographic data, initial operative time, hospital length of stay (LOS), and number and duration of subsequent surgeries were recorded.

**Results:**

In total, 523 referred patients were treated for PJI. The mean initial LOS was 7.0 days (standard deviation [SD] 5.7) and the mean operative time was 169.5 min (SD 75.9). Overall, 36% underwent irrigation and debridement with modular component exchange, single stage revision, or a spacer alone. Of the 335 who had at least 1 subsequent surgery, under half (N = 146, 43.5%), received stage two alone. On average, patients underwent 1.8 subsequent surgeries (SD 1.4), ranging from 1 to 14. The cumulative mean operative time for these subsequent surgeries was 306.4 min (SD 253.3). The number of PJIs nearly doubled from 2014-2017 (N = 112) to 2021-2024 (N = 221). There were no significant changes in LOS, number of subsequent surgeries, or cumulative operative time for reoperations during these intervals.

**Conclusions:**

The volume of referred PJIs has increased substantially over the past decade at our referral center, underscoring the growing demand for PJI care and profound resource burden necessary to care for these patients.

## Introduction

Periprosthetic joint infection (PJI) remains a catastrophic complication following total knee arthroplasty (TKA) and total hip arthroplasty (THA) [1], [2]. According to the most recent report from the American Joint Replacement Registry, PJI was the most common diagnosis for revision THA (26.3%) and revision TKA (32.5%) [3]. Despite significant attention and research on PJI prevention, there has not been a substantial improvement in rates of PJI [4]. As such, the overall volume of PJI is anticipated to increase as the number of primary THAs and TKAs continues to rise.

The management of PJI benefits from a multidisciplinary approach that is not feasible in all healthcare settings and practices [5], [6], [7]. Additionally, the care for these patients is also quite costly, with an estimated annual cost of PJI care in the United States alone at over $1.6 billion [8]. Recently, there has been interest in the creation of specialized centers for the treatment of PJI, much like the well-established concept of specialized centers in oncology [9], [10], [11]. However, in the current climate of decreasing reimbursement in total joint arthroplasty, concern exists among centers about the financial implications of being a referral institution for PJI care as these cases are more complex and require more operative time and resources [12].

Among high-volume centers, the evolution of PJI referrals and growing resource utilization have not been well documented in the literature. This information is critical to inform stakeholders regarding resource management and reimbursement implications for the complex care required in the treatment of PJI. Therefore, the aim of this study was to characterize the evolving resource burdens of PJI referrals at a single high-volume, academic tertiary referral center over the past decade.

## Material and methods

A retrospective review was conducted on all referred patients who underwent surgical intervention for hip or knee PJI at a single, academic tertiary referral center from January 1, 2014, to December 31, 2024. Institutional review board approval was obtained prior to conducting chart review. Epic Slicer Dicer (Epic Systems Corporation, Verona, WI, USA) was utilized to identify all patients who received surgical intervention for PJI. All identified patient charts were thoroughly reviewed to determine whether patients had their primary THA or TKA at our academic center or at an outside institution. Given the aims of our study, only patients who had a primary THA or TKA at an outside institution were included. The diagnosis of PJI was confirmed according to the 2018 International Consensus Meeting criteria. The diagnosis was established and confirmed at our institutions in all cases, rather than accepted from the referring institutions. Specifically, serum erythrocyte sedimentation rate, C-reactive protein, preoperative aspiration, the presence of sinus tracts, and a minimum of 5 separate intraoperative tissue cultures at time of surgical intervention were utilized. Microbiologic and clinical records from the referring institution were reviewed when available and were used to inform antimicrobial management but were not used in isolation to establish the diagnosis.

The study population comprised both inpatient transfers and outpatient referrals. Overall, 272 (52%) patients were accepted as direct inpatient transfers from an outside hospital, while 251 (48%) were evaluated and scheduled electively through our outpatient arthroplasty clinic. All included patients, irrespective of referral pathway, underwent their index arthroplasty at an outside institution and subsequently underwent surgical management of their PJI at our center.

Demographic data were obtained on each patient and include age, sex, body mass index, Charlson Comorbidity Index, and whether the surgery involved the hip or knee, Table 1. Patient demographics were also compared based on time period with the historical cohort being 2014 to 2017 and the recent cohort being 2021 to 2024, Table 2. These two 4-year periods were selected a priori to represent the beginning and the end of the study decade, providing nonoverlapping intervals of equal duration at the temporal extremes of the cohort while preserving an adequate sample size within each group. The intervening years, 2018 through 2020, were deliberately excluded from this paired comparison in order to maximize the temporal contrast between the 2 groups and to avoid the confounding influence of the COVID-19 pandemic, during which the deferral of elective arthroplasty, restrictions on interhospital transfer, and constrained inpatient capacity substantially disrupted both referral volume and length of stay (LOS). It should be emphasized that patients treated between 2018 and 2020 remain included in the overall cohort of 523 patients and in the year-by-year analyses presented in Figure 1, Figure 2, Figure 3, Figure 4, Figure 5. These years were omitted only from the paired historical vs recent comparison. The overall number of PJI referrals was reported for each year, in addition to initial operative time, hospital LOS, and number and duration of subsequent surgeries. This data were also stratified and compared based on time period (historic vs recent). Means and standard deviations (SDs) were reported, and linear regressions were performed for continuous variables. Depending on data type, *Chi-*square, Fisher exact, *t*-tests, and Mann–Whitney *U* tests were performed to determine differences between time periods. Statistical analyses utilized SAS 9.4 (Statistical Analysis Software, Cary, North Carolina), and significance was set at *P* < .05.

**Table 1:** Overall patient demographics.

Demographic	Mean (SD)
Age	65.0 (11.1)
BMI	32.3 (8.4)
CCI	2.3 (2.8)
ASA	2.9 (0.7)
Sex	
Female	253 (48.4)
Male	270 (51.6)

BMI, body mass index; CCI, Charlson Comorbidity Index; ASA, American Society of Anesthesiologists Score.

**Table 2:** Change in patient demographics over time.

Demographic	2014 to 2017 (N = 112)	2021 to 2024 (N = 222)	*P* value
Age	64.2 (10.4)	65.9 (11.6)	.1905
BMI	32.6 (8.5)	32.2 (8.3)	.7099
CCI	2.4 (2.8)	2.2 (2.8)	.4979
ASA	2.8 (0.7)	2.9 (0.7)	.3475
Sex			
Female	64 (57.1)	93 (41.9)	.0084
Male	48 (42.9)	129 (58.1)	
			

BMI, body mass index; CCI, Charlson Comorbidity Index; ASA, American Society of Anesthesiologists Score.

**Figure 1:**
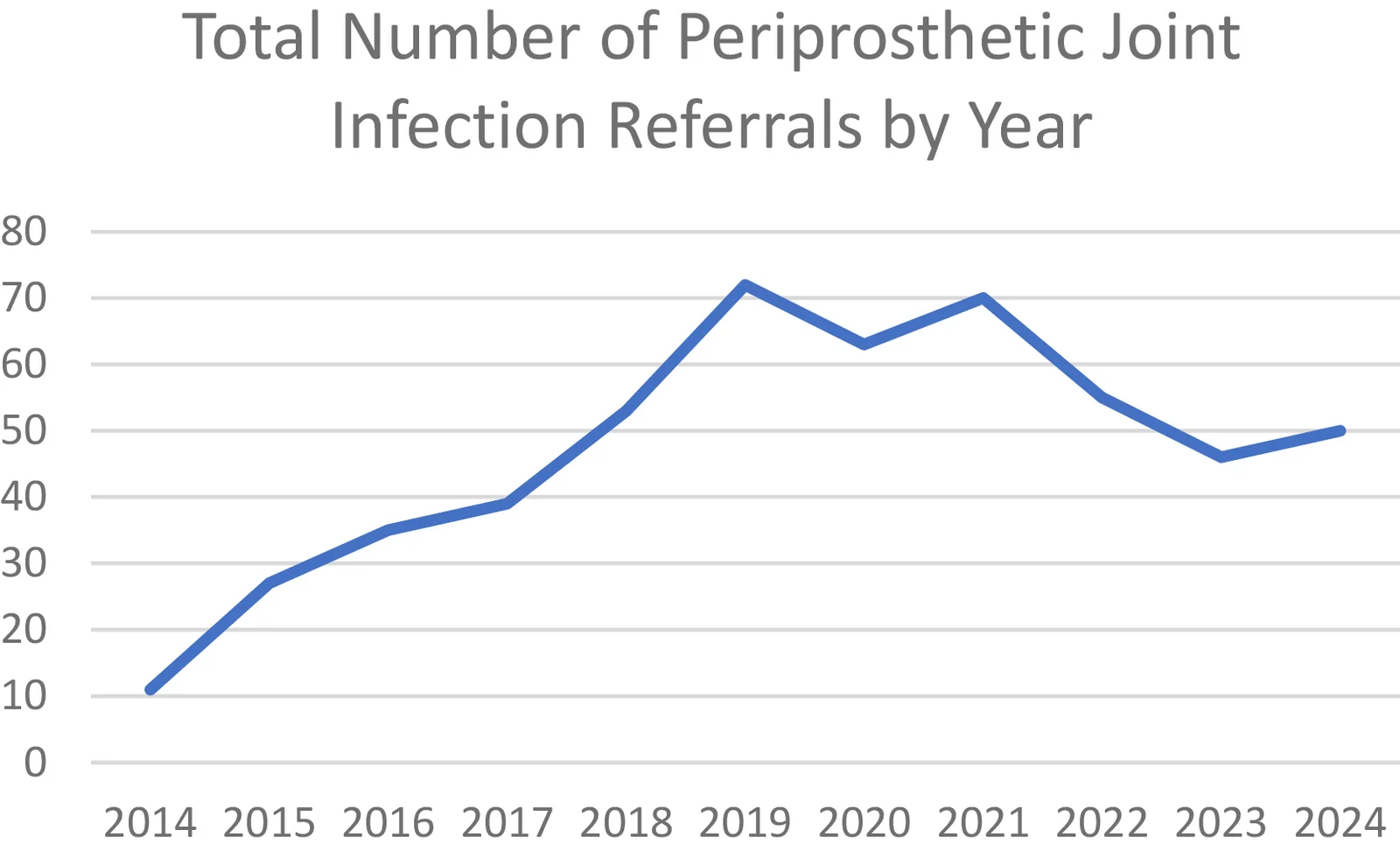
Number of PJI referrals over time.

**Figure 2:**
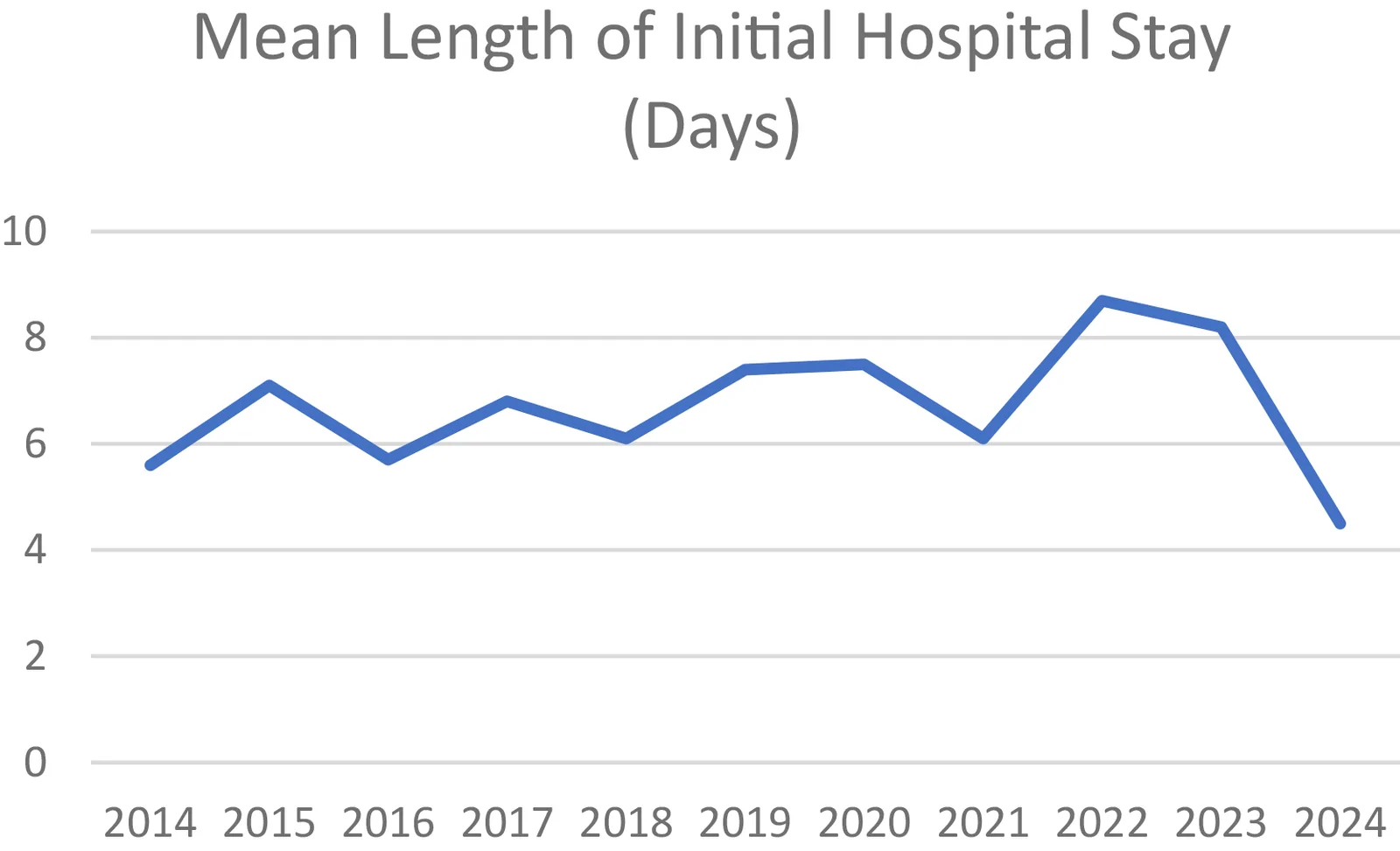
Mean length of initial hospital stay over time.

**Figure 3:**
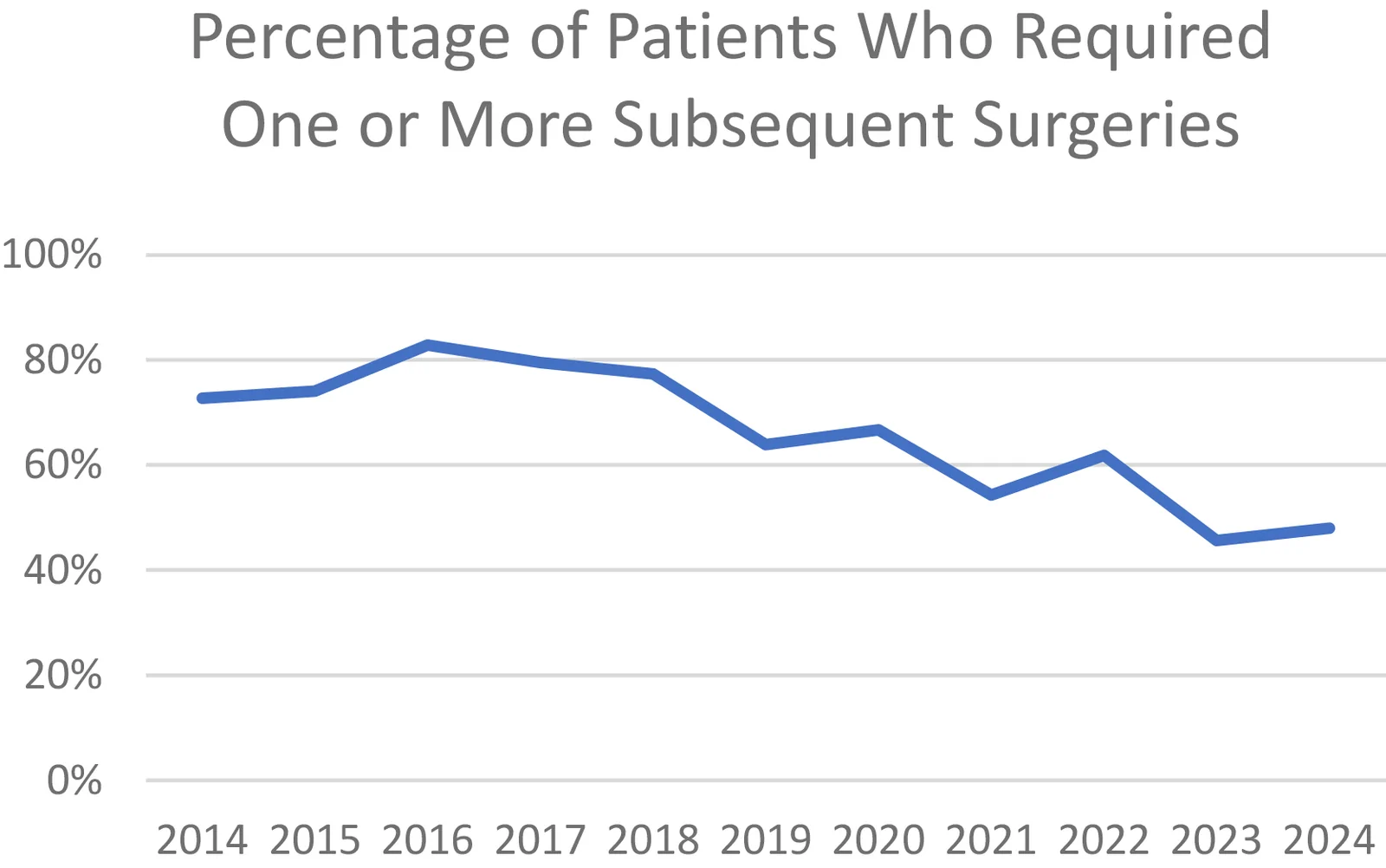
Percentage of patients who required one or more subsequent surgeries.

**Figure 4:**
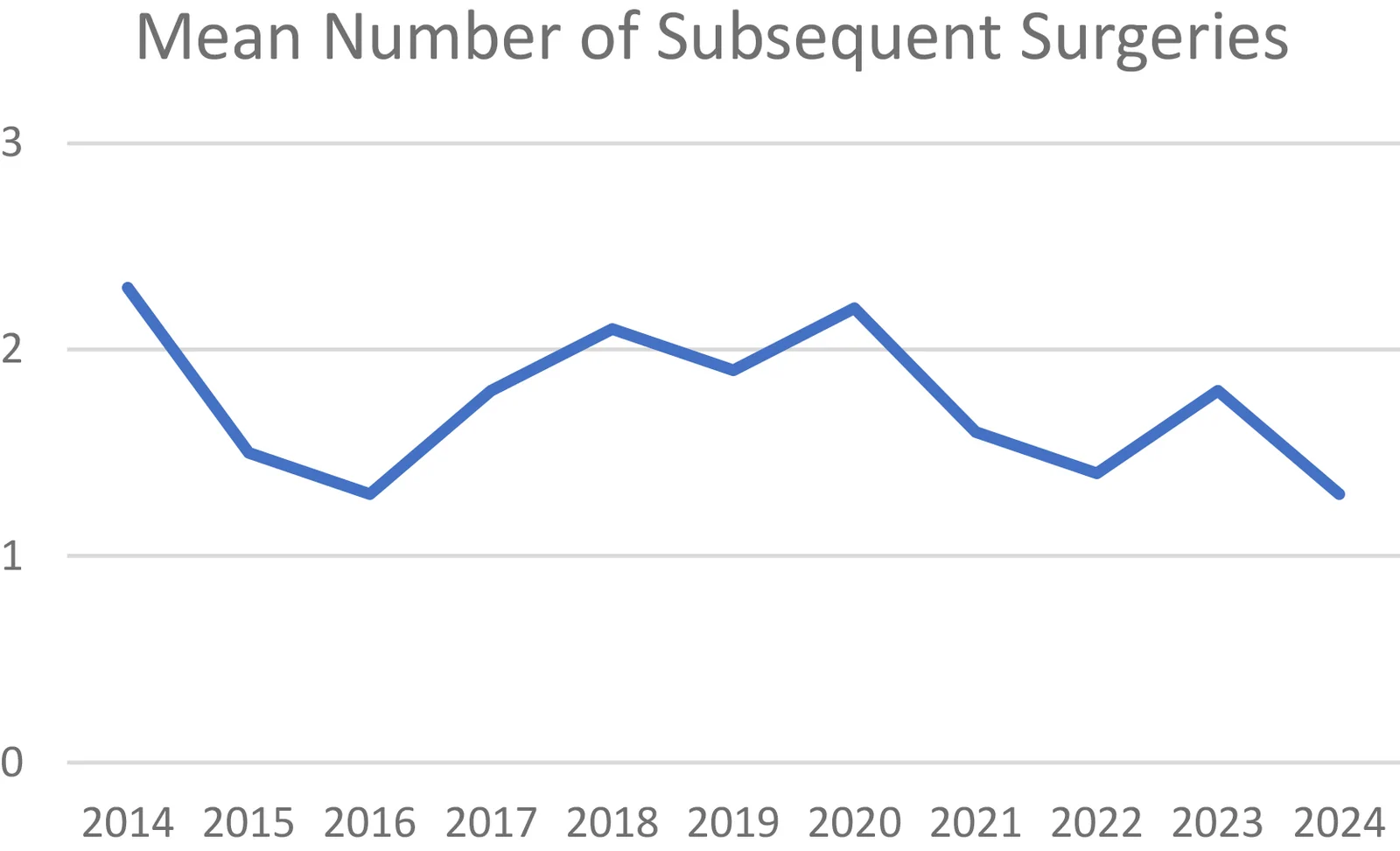
Mean number of subsequent surgeries over time.

**Figure 5:**
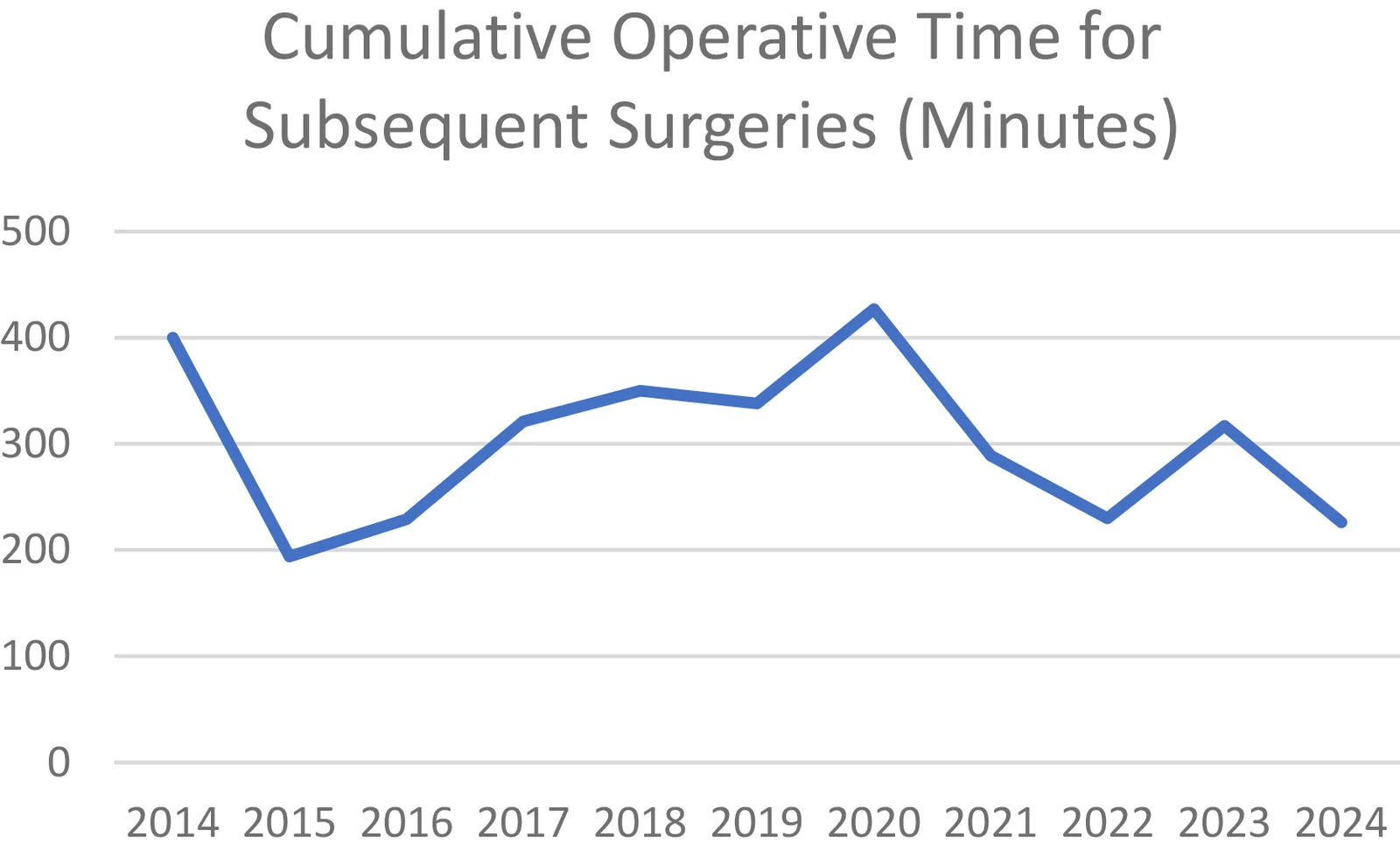
Cumulative operative time for subsequent surgeries over time.

## Results

A total of 523 patients were treated for PJI from 2014 to 2024. The mean age of these patients was 65.0 years (SD 11.1 years), the mean body mass index was 32.3 (SD 8.4), and the mean Charlson Comorbidity index was 2.3 (SD 2.8). Women accounted for 48.4% of referred PJI cases at our institution. Table 2 describes the evolving patient demographics between the historical and recent cohorts. There were significantly fewer women in the recent cohort compared to the historical cohort (41.9% vs 57.1%, *P* = .0084). Otherwise, there were no differences in demographics between the historical and recent groups.

Of the total 523 patients, 335 (64.1%) required at least 1 additional surgical procedure (Table 3). Of the 523 patients, 36% underwent irrigation and debridement with modular component exchange, a single stage revision, or a spacer alone. Of the 335 that had at least 1 subsequent surgery, under half (N = 146, 43.5%), received a second-stage revision alone. Overall, 64% required more than 1 subsequent surgery after their initial operation (which was usually a spacer).

**Table 3:** Overall burden of PJI care.

Burden characteristic	N (%)
Had at least 1 subsequent surgery	335 (64.1%)
Length of initial hospital stay (days)	7.0 (5.7)
Operative time of initial surgery (minutes)	169.5 (75.9)
Number of subsequent surgeries	1.8 (1.4)
	Mean (SD)
Operative time for subsequent surgeries (minutes)	306.4 (253.3)

The mean initial hospital LOS was 7.0 days (SD 5.7), and the mean operative time for the index surgery was 169.5 min (SD 75.9). On average, patients underwent 1.8 subsequent surgeries (SD 1.4), ranging from 1 to 14 additional surgeries. The cumulative mean operating room (OR) time for these subsequent surgeries was 306.4 min (SD 253.3) (Table 3). Comparing referral trends over time, the number of PJI cases nearly doubled from 2014 to 2017 (n = 112) to 2021-2024 (n = 221). Despite this increase in volume, there were no statistically significant changes in hospital LOS, number of subsequent surgeries, or cumulative operative times between these time intervals, (Table 4). Table 5 provides a summary of the trends in resource burden of PJI care over time. Figure 1, Figure 2, Figure 3, Figure 4, Figure 5 visually demonstrate the evolving burden of PJI care at our institution.

**Table 4:** Change in burden of PJI care over time.

Burden characteristic	2014 to 2017 (N = 112)	2021 to 2024 (N = 221)	*P* value
	N (%)	N (%)	
Had at least 1 subsequent surgery	88 (78.6)	117 (52.2)	<0.001
	Mean (SD)	Mean (SD)	
Length of initial hospital stay (days)	6.4 (4.5)	7.6 (6.7)	0.0595
Operative time of initial surgery (minutes)	151.8 (70.2)	174.6 (77.0)	0.0092
Number of subsequent surgeries	1.6 (1.2)	1.5 (1.1)	0.5171
Operative time for subsequent surgeries (minutes)	269.0 (209.7)	264.4 (229.0)	0.8818

**Table 5:** Overall trends in burden of PJI care over time.

Burden characteristic	2014 (N=11)	2015 (N=27)	2016 (N=35)	2017 (N=39)	2018 (N=53)	2019 (N=72)	2020 (N=63)	2021 (N=70)	2022 (N=55)	2023 (N=46)	2024 (N=50)	*P* value
	n (%)	n (%)	n (%)	n (%)	n (%)	n (%)	n (%)	n (%)	n (%)	n (%)	n (%)	
Had at least 1 subsequent surgery	8 (72.7)	20 (74.1)	29 (82.9)	31 (79.5)	41 (77.4)	46 (63.9)	42 (66.7)	38 (54.3)	34 (61.8)	21 (45.7)	24 (48.0)	<0.0001
	Mean (SD)	Mean (SD)	Mean (SD)	Mean (SD)	Mean (SD)	Mean (SD)	Mean (SD)	Mean (SD)	Mean (SD)	Mean (SD)	Mean (SD)	
Length of initial hospital stay (days)	5.6 (3.2)	7.1 (6.1)	5.7 (2.8)	6.8 (4.8)	6.1 (3.3)	7.4 (6.7)	7.5 (6.9)	6.1 (3.3)	8.7 (5.9)	8.2 (9.0)	4.5 (0.7)	0.0271
Operative time of initial surgery (minutes)	164 (57)	159 (67)	154 (66)	178 (82)	185 (72)	189 (81)	206 (86)	201 (68)	199 (82)	180 (73)	208 (72)	0.0004
Number of subsequent surgeries	2.3 (1.8)	1.5 (0.7)	1.3 (0.5)	1.8 (1.6)	2.1 (2.2)	1.9 (1.3)	2.2 (1.8)	1.6 (1.1)	1.4 (0.7)	1.8 (1.5)	1.3 (0.9)	0.0002
Operative time for subsequent surgeries (minutes)	400 (399)	194 (93)	229 (110)	321 (247)	350 (264)	338 (259)	427 (337)	289 (236)	230 (114)	317 (356)	226 (196)	0.7535

## Discussion

This study demonstrated a substantial increase in the volume of referred PJI cases treated at our high-volume tertiary referral center over the past decade. Despite almost doubling the number of PJI referrals from 2014 to 2017 and 2021 to 2024, the hospital LOS, number of subsequent surgeries, and cumulative reoperation time remains consistently high without significant changes over time. More than 60% of referred patients required at least one further reoperation, but the range of additional surgeries varied substantially from 1 to 14 additional procedures. The substantial variability likely reflects the heterogeneity of the PJI population rather than variability in surgical technique. Host factors are a principal determinant, such as those with poorly controlled diabetes, immunosuppression, chronic kidney disease, vascular disease, or a compromised soft tissue envelop are less able to clear infection and correspondingly more likely to require repeat interventions. Microbiologic factors are equally influential, as difficult-to-treat organisms and antibiotic-resistant infections are well-recognized to portend inferior outcomes and greater likelihood of repeat surgery [13]. On average, the cumulative mean reoperation time for these subsequent surgeries was 5 hours, although the SD for this group was greater than 4 hours. These findings underscore the intensive resource utilization required for adequate PJI care and reflect the complex treatment course associated with PJI.

As hospital LOS has decreased in both primary and revision TKA and THA, one could reasonably expect a downward trend in the LOS for patients who suffer from PJI [14]. However, this was not observed in our institutional review of PJI care. Contributing factors to this maintained high LOS include an increasingly complex cohort of patients referred to academic institutions [15]. Additionally, inherent care pathways may contribute to this sustained high LOS, which include inpatient infectious disease consults and the reliance on cultures to direct antibiotic therapy, often taking several days to result [16].

Similarly, the time required for the surgical care of these patients has not declined. As shown, the amount of time on subsequent surgeries is substantial, highlighting the complexity of these cases. Many of these subsequent surgeries are 3 to 5 hours long, and longer than the index revision. While one could postulate that with increasing volume over time, familiarity and repetition would lead to reduced surgical time. However, the finding that our surgical time has not decreased over time highlights that these cases are not routine and do not lend themselves to speed or efficiency. Instead, the surgeries are highly complex, requiring significant personnel and resources. Patients referred from an outside system have, by definition, already progressed beyond the point at which their treating institution felt able to manage them, and referral selects for chronic, indolent, or previously treated disease rather than for acute postoperative infection amenable to debridement and implant retention. Many patients arrive with retained hardware, a previously placed spacer, prior failed debridement, established sinus tracts, or extensive bone loss. We believe this selection bias to be the primary driver of continued case complexity at our center for PJI care. Additionally, evolving practices aimed at improving outcomes, such as double draping to reduce infectious contamination and bioburden of the surgical field, may also explain additional time required for these cases [17].

As these findings are confined to a single institution, it is helpful to compare them to external reference points. However, published benchmarks for resource utilization in PJI care remain limited. Our mean initial LOS of 7 days is nearly identical to the 7.6 days reported by Kapadia et al. [18] for hip PJI managed at a specialized tertiary care center. Both of these LOS are more than double than those reported for aseptic revision arthroplasty in contemporary series [14], [18]. Operative time is substantially more difficult to benchmark, given the limited reporting in PJI series. For context, Panwar et al. [19] prospectively recorded mean operative time of 118 min for revision knee arthroplasty and 150 min for revision THA, which are both shorter than the mean initial operative time of 169.5 min in our infected cohort and substantially shorter than the cumulative 306.4 min of subsequent operative time our patients ultimately required. Taken together, these comparisons suggest that the resource burden we observed is unlikely to be unique to our institution, and we believe future collaboration is necessary to meaningfully improve the high demands to successfully and efficiently care for these patients.

There are additional burdens to the care team outside of direct patient care for the treatment of PJI that we were unable to capture in our data. For example, Mohler et al. performed a retrospective review of perioperative electronic health record activity for over 175 patients who suffered from hip or knee PJI [20]. The authors noted a high level of administrative burden required in the surgical management of PJI, with over 8 hours of administrative work typically required. They also noted that surgeons alone performed more than 450% more administrative work for PJI during the preoperative period compared to primary total joint arthroplasty.

As it relates to healthcare costs, multiple studies have evaluated the effectiveness of single- vs two-stage revision for the management of chronic PJI [13], [21], [22], [23], [24], [25], [26], [27], [28]. As such, the enthusiasm for single-stage revision for chronic PJI is growing, which has implications for overall cost to the healthcare system since a second surgery is not anticipated. For example, over 40% of subsequent surgeries could have been eliminated in our cohort if planned second-stage revisions became obsolete. However, single-stage revision for chronic PJI require intensive intraoperative resources and surgeon time, which has historically been poorly funded. The intraoperative demands of single-stage revision warrant particular attention, because the appeal of eliminating a second procedure obscures the extent to which the burden of that procedure is relocated rather than removed. A single-stage exchange requires radical debridement of all infected bone and soft tissue, complete removal of all implants and cement, and definitive reconstruction, all within a single operation. In practice, this typically entails 2 entirely separate surgical setups, with redraping, regowning, regloving, and a second complete instrument set introduced following the debridement phase, together with prolonged anesthesia time. From the standpoint of the OR, a single-stage revision is a longer and more demanding operation, and it is reimbursed under a single global period. In response, Fehring et al. [29] evaluated the reimbursement and operative time for 50 single-stage revisions for hip and knee PJI compared to 250 primary TKAs and THAs by the same surgeons. For knees, the mean hourly compensation for a single-stage revision was $601.60, while the mean hourly compensation for a primary total knee was $1461.00. For hips, the mean hourly compensation for a single-stage revision was $545.624, while the mean hourly compensation for a primary total hip was $1589.26. The authors encouraged payers to consider appropriate reimbursements for single-stage procedures to both decrease patient morbidity and ultimately lower healthcare systems costs as a whole.

There are several limitations to this study that should be addressed. Among these is that this is a single institution study, reflecting referral patterns, surgical practices, and institutional care pathways of 1 academic tertiary referral center. We are unable to conclude if these findings are generalizable to other institutions. The retrospective nature introduces inherent limitations, which includes the potential for selection bias as we were only able to assess patients who ultimately underwent surgical intervention at our academic center. There is a possibility that, due to staffing or bed shortages, the actual number of surgically treated referrals is an underestimate of the potential PJI cases that were attempted to be referred to our institution. Additionally, while we examined hospital LOS and operative time as indicators of resource utilization, our analysis was unable to account for all potential costs, including outpatient follow-up, intravenous medications, home-nursing care, among others. We were also unable to completely characterize the chronicity of infection at presentation, and specifically the interval between the index arthroplasty and the onset of infection. Because acute postoperative and acute hematogenous infections differ substantially from chronic infections in both their management and their expected reoperation burden, the absence of this variable limits our ability to explain the heterogeneity we observed in the number of subsequent procedures. Finally, while we observed a temporal change in PJI volume at our institution when comparing a historical to a recent cohort of PJI referrals, our retrospective study was not designed to evaluate causative factors to explain these trends.

Several specific future directions follow from this study’s limitations. Multi-institutional studies incorporating both high-volume referral centers and lower-volume community institutions will be necessary to determine whether operative times, LOS, and reoperation rates reported here are universal or center-specific. This would also allow identification of institutions that have successfully streamlined aspects of PJI care, along with the responsible care pathways. Formal economic analyses employing time-driven activity-based costs, rather than LOS and operative time as surrogates, would provide a more accurate accounting of the true cost of an episode of PJI care and of the degree to which current reimbursement fails to meet it. Finally, direct comparison of outcomes and resource utilization between referral and nonreferral centers treating comparable patients would help establish whether the concentration of PJI care at specialized centers delivers its presumed value.

## Conclusions

Over the past decade, our institution has experienced a marked increase in referrals for PJI, which parallels the growing volume of primary TKAs and THAs being performed. As a high-volume tertiary referral center, we have absorbed a high amount of resource burden associated with managing PJI, as reflected by substantial OR time and extended hospital LOS for patients’ initial and subsequent surgeries. This increasing surgical volume and consistently high resource burden of PJI care underscores the need for reimbursement reform to sustain specialized care at high-volume PJI referral centers and to develop much needed multidisciplinary centers of excellence for treatment of these difficult cases.

## Conflict of interest

Joshua Rainey serves on the editorial/governing board for Arthroplasty Today. Christopher L Peters receives royalties from Zimmer Biomet; is a paid consultant for Zimmer Biomet; holds stock or stock options in Kyniska Robotics; receives research support from Zimmer Biomet; serves as a board or committee member for Hip Society. Christopher Pelt receives royalties from Joint Development, Peptilogics, Zimmer Biomet; receives honoraria for speakers bureau activities or paid presentations for Total Joint Orthopaedics, Solventum; is a paid consultant for Solventum, Total Joint Orthopaedics; receives research support from Zimmer Biomet; serves on the editorial/governing board for Journal of Arthroplasty, Arthroplasty Today; serves as a board or committee member for AAHKS, Chair Publications Comittee. All other authors declare no potential conflicts of interest.

For full disclosure statements refer to https://doi.org/10.1016/j.artd.2026.102196.

## CRediT authorship contribution statement

**Joshua P. Rainey:** Writing – review & editing, Writing – original draft, Methodology, Investigation, Data curation, Conceptualization. **Brenna E. Blackburn:** Writing – review & editing, Writing – original draft, Methodology, Formal analysis, Data curation. **Reed C. Noyd:** Investigation, Data curation. **Jessica Hammond:** Methodology, Investigation, Data curation. **Laura K. Certain:** Writing – original draft. **Michael J. Archibeck:** Writing – original draft. **Lucas A. Anderson:** Writing – original draft. **Christopher L. Peters:** Writing – review & editing, Writing – original draft, Supervision, Conceptualization. **Jeremy M. Gililland:** Writing – review & editing, Writing – original draft, Methodology, Conceptualization. **Christopher E. Pelt:** Writing – original draft.

## Appendix A Supplementary data

Conflict of Interest Statement for Anderson

Conflict of Interest Statement for Archibeck

Conflict of Interest Statement for Blackbur

Conflict of Interest Statement for Certain

Conflict of Interest Statement for Gilillande

Conflict of Interest Statement for Hammond

Conflict of Interest Statement for Noyd

Conflict of Interest Statement for Pelt

Conflict of Interest Statement for Peters

Conflict of Interest Statement for Rainey
