# Supplementary material for: A Decade in Periprosthetic Joint Infection Care: Growing Referrals and High Resource Demands at a High-Volume Center
Source: Arthroplast Today. 2026 Oct 28;42:102196. doi: 10.1016/j.artd.2026.102196 (PMC13738014; doi:10.1016/j.artd.2026.102196)
Supplement: Supplementary file 6 [file mmc4.docx]

# INDIVIDUAL CONFLICT OF INTEREST STATEMENT

***American Association of Hip and Knee Surgeons***

(Adopted from the American Academy of Orthopaedic Surgeons disclosure statement)

The following form **must be filled out completely and submitted by each author (example, 6 authors, 6 forms).**

**All items require a response. If there is no relevant disclosure for a given item, enter "*None*.”**

**Manuscript Title:** A Decade in Periprosthetic Joint Infection Care: Growing Referrals and High Resource Demands at a High-Volume Center

1.	Royalties from a company or supplier (The following conflicts were disclosed)

None

2.	Speakers bureau/paid presentations for a company or supplier (The following conflicts were disclosed)

One-time paid speaker on a panel sponsored by BoneSupport

3A.	Paid employee for a company or supplier (The following conflicts were disclosed)

None

3B.	Paid consultant for a company or supplier (The following conflicts were disclosed)

Paid consultant for ZimmerBiomet and 3M

3C.	Unpaid consultants for a company or supplier (The following conflicts were disclosed)

Unpaid consultant for Osteal

4.	Stock or stock options in a company or supplier (The following conflicts were disclosed)

None

5.	Research support from a company or supplier as a Principal Investigator (The following conflicts were disclosed)

None

6.	Other financial or material support from a company or supplier (The following conflicts were disclosed)

None

7.	Royalties, financial or material support from publishers (The following conflicts were disclosed)

None

8.	Medical/Orthopaedic publications editorial/governing board (The following conflicts were disclosed)

Subject editor for *Foot and Ankle International* and *Foot and Ankle Orthopaedics*

9.	Board member/committee appointments for a society (The following conflicts were disclosed)

Executive Board member of MSIS, 2020-2025

**Each author must sign AND print or type his/her name, date and submit a separate form**

In addition, one BLINDED Conflict of Interest form (no author names used) should be submitted per manuscript with all author disclosures.

Laura Certain, MD, PhD 11/16/2025

Author Name (Print or Type)	Author Signature	Date
